# Masculinities and suicide: A systematic review and meta-analysis

**DOI:** 10.1371/journal.pone.0342172

**Published:** 2026-02-25

**Authors:** Charlotte Starkey, Fhionna Moore

**Affiliations:** 1 School of Social Science, Humanities, and Law, University of Dundee, Dundee, United Kingdom; 2 Department of Psychological Services, NHS Tayside, Perth, Perth and Kinross, United Kingdom; Universidade Federal do Rio Grande do Sul, BRAZIL

## Abstract

**Background:**

Women are more likely to report suicidal ideation and make suicide attempts, while men are more likely to die by suicide. There has been much discussion of the possible contribution of masculinity (i.e., attitudes, beliefs, and behaviours understood to be ‘masculine’ through construction of gendered identities in local contexts) to this gender paradox of suicide. Here we report the first systematic literature review, meta-analysis, and meta-regression testing relationships between measures of masculinity and suicidality.

**Methods:**

We searched for articles using the following search terms in Google Scholar, Web of Science, PubMed, and APA PsychINFO in July 2024 ((“gender role*” OR “gender-role*” OR “sex role*” OR “sex-role*” OR “masculin*” (TOPIC)) AND (“suicid*” (TOPIC))). We excluded papers which examined non-suicidal self-injury, were not based on individual-level valid and reliable quantitative measures of masculinity and suicidality, and which did not provide sufficient statistical information to compute effect size.

**Results:**

Across 23 studies the relationship between the multiple and diverse measures of masculinity and of suicidality overall was non-significant (r = 0·03 [95% CI: −0·01, 0·1], z = 1.75, p = 0.341) and showed significant heterogeneity. Given the range of operationalisations of masculinity included, and the high heterogeneity, we urge caution in interpreting the pooled overall relationship. Meta-regression showed moderation of the overall relationship by both measure of masculinity and suicidality. That is, the positive relationship between masculinity and suicidality was stronger for measures of masculinity that focussed on emotional restriction and pursuit of status, and for suicidal action than ideation. Conversely, there were inverse relationships between strength-based measures of masculinity and suicidality.

**Limitations:**

Our results demonstrated significant heterogeneity, and measures of masculinity are likely to be largely outdated.

**Conclusions:**

Our results suggest there is value in further work identifying specific aspects of contemporary psychological masculinity which link to suicidality, which is of relevance to clinical assessment and management of suicidality.

## Introduction

Worldwide, men are more likely than women to die by suicide [1,2]. According to the World Health Organisation (2017) [3], this is at a rate of 1.8 times as many deaths by suicide amongst men as women. The magnitude of this sex difference, however, is not stable. It is greatest in high income countries, and reversed in others (e.g., China and Morocco; [3]). Furthermore, women are more likely to report suicidal thoughts and to make non-fatal suicide attempts (the ‘gender paradox’ of suicide [1,4–8]). Therefore, much of the discourse around sex, gender, and suicide, has sought to understand the ways in which cultural and societal pressures on men and women impact upon behaviours, attitudes, and beliefs around suicidality (defined here as the full spectrum of suicidal thoughts and behaviour, including suicidal ideation, suicide attempts, and death by suicide, but not including non-suicidal self-harm).

‘Sex’ is meant here as the labelling of an individual as male or female based on biological characteristics at birth, and gender as prevailing norms of masculinity and femininity in a particular time and place [9]. The link between sex and gender is through the ways in which culture enforces scripts of what it means to be male and female through rules, laws, and norms [10–12]. As such, attitudes, beliefs, and behaviours become understood to be ‘masculine’ or ‘feminine’ through construction of gendered identities in local contexts [11,13]. The ‘perfect’ masculine ideal in a particular society, is an amalgamation of all the ‘right’ [14–16] masculine traits necessary to succeed in the male social role within a gendered social structure. This means there is no single universal masculine or feminine ideal, and that masculinity and femininity are concepts which adapt and mould flexibly to fit cultures and social settings [14–17].

Defining and operationalising masculinity (or masculinities), then, is not straightforward and a universal definition is unlikely to be meaningful. The vast majority of quantitative research into links between masculinities and suicidality, however, has been conducted on westernised high-income populations, in which the gender paradox of suicide is most pronounced. Here, masculinity has been described by Houle et al., (2008) [18] as consistent with Jansz’s (2000) [19] definition of four attributes of the ‘traditional’ male gender role: success, autonomy, stoicism, and aggression. Coleman et al., (2011) [20] expands this to include the traits of competitiveness, strength, emotional avoidance, avoidance of being perceived to be feminine, and being action-orientated. They argue that these traits are linked with cognitive rigidity, meaning they are brittle and do not allow for flexible adaptation to adverse circumstances resulting from life events [20]. Traits of this traditional western masculinity, then, may promote suicidality by reducing capacity to cope with adversity. It has been argued that this is exacerbated by increased experience of adversity by those who adhere to traditional norms in societies with growing gender equality and a movement away from traditional western gender roles [21,22]. For example, men may experience perceived or actual reduction in role opportunities under increasing gender equality [21], or may experience role conflict in which they attempt to meet the expectations of traditional roles to be met with negative social responses [22]. ‘Traditional masculinity’ will be used throughout the rest of this review to refer to the specific masculinity of high-income western countries as described above. We acknowledge, however, that this is likely to be a blunt operationalisation of a complex concept encompassing multiple masculine constructs, which vary across contexts, and that alternative masculinities exist (both to different regions, and alternative versions within regions) and are likely to relate differently to suicide.

It is possible to predict a number of links between traditional masculinity and health outcomes including suicide [22–24]. The detrimental impact of traditional masculinity on help-seeking [25] and emotional expression [26], greater impulsivity [27] and misuse of alcohol [28] amongst men than women, and greater social acceptability of more lethal methods of suicide for men [29], for example, are all pathways by which psychological and behavioural masculinity may increase risk of death by suicide. Men who struggle to live up to traditional masculine ideals may experience shame [30]. For example, men expressing emotion in a way consistent with the female gender role are perceived as signalling subordination, and are policed by other men [30], leading to restricted emotional expression and a reluctance to report or demonstrate distress [31].

It is also possible, however, that broad traditional masculinity encompasses traits that are protective against suicidality [32,33], such as providing for a family and wanting to be a good father [32]. Oliffe et al., (2011) [34], for example, quote one participant as saying: “*I’ve got to feed the wife and kids, I’ll go to work anyway even though life sucks*” (p. 449). Emslie et al., (2006) [35] noted how all the male participants in their study who had thought about suicide had been deterred by thoughts of the grief they would be inflicting on their family and friends. The role of fatherhood here, however, is also complex with some research showing parenthood to be a significant protective factor in the suicidal process only for women [36], and others showing men who have been denied access rights to their children following the breakdown of a relationship with the mother as being at high risk of suicidality [37].

Finally, it is important to recognise the ways that traditional masculinity intersects with other characteristics. For example, potential effects of traditional masculinity on suicidality are not limited to men. While women, as a group, exhibit lower levels of traditional masculinity overall, they are not exempt from possessing masculine traits [38]. We anticipate that there may be complex interactions between sex and masculinity. Furthermore, suicide rates vary in relation to age [39], rural versus urban location [40,41], and sexual orientation [42]. Therefore, associations between traditional masculinity and suicidality must take into consideration the personal and local context of the individual.

To summarise, there are theoretical justifications for both positive and negative impacts of traditional masculinity on suicidality which may contribute to the gender paradox of suicide. At first glance, the existing literature on relationships between masculinity and suicidality is complex, without definitive answers [43], perhaps due to the wide variety of ways in which masculinity has been operationalised and measured. There has to date been no attempt to draw this literature together to crystallise these relationships through meta-analysis, or to quantify the ways in which masculinity may intersect with personal or contextual characteristics. Therefore, our research questions are as follows:

### RQ1: Are measures of traditional masculinity correlated with suicidality?

Bearing in mind the diverse ways in which masculinity is measured in the literature, we anticipate the possibility that the answer to RQ1 may be multifaceted, and therefore we propose the following questions in order to crystallise patterns between specific operationalisations of traditional masculinity and suicidality:

### RQ2: Do different operationalisations of traditional masculinity (or components of traditional masculinity) show different relationships (in terms of direction and magnitude) with suicidality?

**RQ3: Do measures of traditional masculinity correlate differently (in terms of direction and magnitude) with suicidal ideation, suicide attempts, and deaths by suicide?**

Finally, we acknowledge the likely intersection between masculinity, personal characteristics, and local context:

**RQ4: Are relationships between traditional masculinity and suicidality moderated by personal characteristics (age, sex, or ethnicity), or local context (country)?**

## Methods

The review methodology and planned analyses were pre-registered with PROSPERO (registration number CRD42023430253) and were conducted in accordance with PRISMA guidelines ([44]; please see Appendix A for completed PRISMA Checklist).

### Search strategy

We searched for quantitative studies which tested relationships between masculinity or male gender/sex role and suicidality in the following databases: Google Scholar, Web of Science, PubMed, and APA PsychINFO. Searches were for combinations of terms relating to masculinity and gender- or sex-roles and suicidality, and were conducted in July 2024 (see Appendix B for search strategies). In addition, we searched journals likely to publish articles on masculinity and suicide (Archives of Suicide Research, International Journal of Men’s Health, Psychology of Men and Masculinity, American Journal of Men’s Health, Journal of Men’s Health, Sex Roles, and Suicidological Research Online), and carried out forward and backward searching on the articles included in the first round of screening. We included grey literature in our searches (including dissertations and theses) in our searches.

### Inclusion criteria

**Fig 1:**
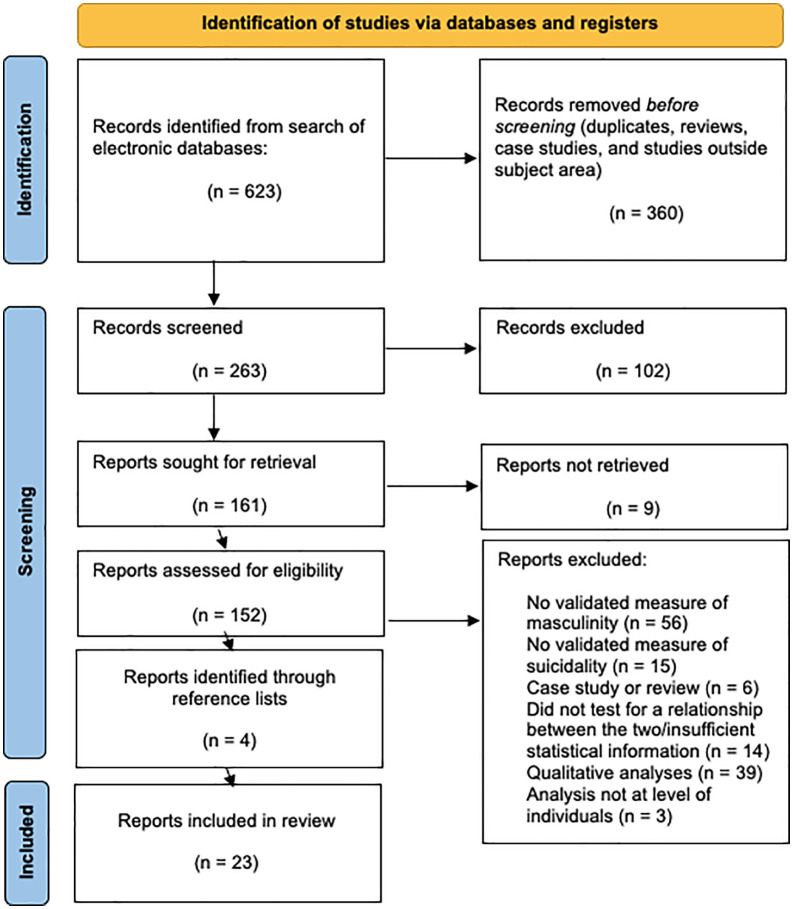
PRISMA flowchart of study selection.

Studies were included if they met the following criteria (see Fig 1):

1. Tested relationships between individual-level masculinity and suicidality

As our aim was to determine if, and how, measures of masculinity correlate with suicidality in the lives of individuals, we included only those studies which assessed links between the two at the level of individuals. We did not include studies which assessed relationships between national or regional proxies of masculinity in relation to population-level suicide rates.

2. Reported valid and reliable quantitative measures of psychometric masculinity

We included only those studies which utilised measures of masculinity for which validity and reliability could be established. We focussed here on quantitative analyses to quantify magnitudes of relationships across studies and to test roles of moderators. We acknowledge, however, the valuable body of qualitative work in this area and have referred to this where relevant in discussing our findings.

3. Reported valid and reliable quantitative measures of suicidality

We included only those studies in which suicidality was assessed using valid and reliable measures (e.g., scores on validated tools, or taken from medical or death records). We did not include studies which reported deliberate self-harm where suicidal intent was not specified, or where self-harm was reported to be non-suicidal. While non-suicidal self-harm may be linked to the development of capability for suicide (e.g., [45]), it may also be a distinct behaviour independent of suicidality with independent functions (e.g., distraction from psychological pain (e.g., [46]). Therefore, we included only those studies in which suicidal intent or ideation was indicated to avoid conflating alternative functions of self-harm.

4. Studies contained sufficient statistical information to calculate effect sizes (or this was made available by authors)

Where effect sizes, or information required to calculate these, were not included, we contacted study authors to request this information. If this information was not available, the study was excluded.

5. Studies were in English (or an English language version was made available by the study authors)

Where studies were not published in English, we contacted authors to request an English-language version where available. Where an English-language version was not available, the study was excluded.

We did not exclude studies on the basis of year of publication, location, or research design.

### Data extraction

Studies were screened independently by CS and FM for suitability for inclusion and to extract data. Interrater agreement on decisions whether to include or exclude studies was high (96%) and discrepancies were discussed and resolved to reach 100% agreement.

### Effect sizes

We recorded effect size and sample size reported by authors. Where sample sizes varied by analyses (e.g., if sub-group analysis was conducted), we also recorded analysis-specific sample sizes. Where effect sizes were not reported, but sufficient statistical information was included to allow us to calculate these, we did so. As our research questions concerned relationships between variables, we anticipated that correlation coefficients would be the most commonly reported measures of effect size. For the purpose of meta-analysis, we planned therefore to use measures of Pearson’s r. Where r was not reported, we calculated it from statistical information available in the article, or provided by authors. Where a bivariate association was reported alongside a multivariate association controlling for covariates we extracted statistical information for the multivariate result. Where results were reported both separately for men and women, as well as combined, we extracted data for men and women separately. We followed Cohen’s (1988) [47] classification of small (r = 0.1), medium (r = 0.3), and large (r = 0.5) effect sizes.

### Gender/sex

We calculated percentage of the sample that was female based on reported demographics.

### Location

We recorded the country from which the sample was drawn

### Age

We recorded age profile of the sample from demographic information, through reported measure of central tendency or dispersion.

### Ethnicity

We recorded ethnicity distribution of the sample

### Measure of suicidality

Measure of suicidality was recorded (e.g., name of scale used to measure suicidal ideation or suicide attempts, or cause of death recorded in death reports).

### Measure of masculinity

Measure of masculinity was recorded (e.g., name of scale used).

### Quality assessment

CS and FM each independently assessed articles against inclusion criteria and the Joanna Briggs Checklist for Analytical Cross-Sectional Studies [48]. This checklist of 8 quality criteria assesses validity and reliability of measurements, descriptions of inclusion criteria and subjects, appropriateness of statistical analyses, and identification and management of cofounding variables. It allows robust assessment of methodological quality and potential for bias in cross-sectional studies [48], allowing us to evaluate quality in our sample of cross-sectional studies which investigated relationships between exposures and outcomes. Where there were discrepancies (6% of items), discussions were held and complete agreement was reached.

### Data analysis

To answer RQ1 (‘Are measures of traditional masculinity correlated with suicidality?’) we assessed the pooled weighted effect size across all studies using Hedge’s random effects model (including both subject and sampling error). Effect sizes were standardized using Fisher’s r-to-Z transformation. Where there were multiple results for a single study, the unit of analysis was average effect size per study. Heterogeneity was assessed using I^2^, Cochran’s Q, and confidence intervals. We repeated this analysis for (a) the full sample, (b) with a randomly selected single study removed to assess sensitivity, and (c) with Joanna Briggs Inventory quality assessment scores included as a covariate in order to detect any impact of study quality on the relationship.

To answer RQ2 (‘Do different operationalisations of traditional masculinity (or components of traditional masculinity) show different relationships (in terms of direction and magnitude) with suicidality?’), we carried out meta-regression with measure of masculinity included as a moderator.

To answer RQ3 (‘Do measures of traditional masculinity correlate differently (in terms of direction and magnitude) with suicidal ideation, suicide attempts, and deaths by suicide?’), we carried out meta-regression with measure of suicidality included as a moderator.

To answer RQ4 (‘Are relationships between measures of traditional masculinity and suicidality moderated by personal characteristics (age, sex, or ethnicity), or local context (country)?’), we carried out meta-regressions with age profile of sample, ethnicity, sex/gender, and country, as moderators in turn.

For meta-regressions, all results were included for each study to allow us to detect moderation across multiple measures per study. This is an approach that has been advised against in the past due to non-independence of multiple results from the same study, but is now acceptable as modern meta-regression is robust to this independence if corrected for repeated sampling using the Hartung-Knapp correction [49]. In all cases we also ran the moderation analyses using mean effect size per sample, in order to assess sensitivity.

Publication bias was visualised using funnel plots and assessed using Duval and Tweedie’s Trim and Fill method. All analyses were conducted with Comprehensive Meta-Analysis Version 3 (2014) and IBM SPSS v25.

## Results

The search returned 623 items, of which FM screened titles and removed 370 duplicates, reviews, case studies, and studies that were obviously outside the area of interest (see Fig 1). CS and FM screened the abstracts of the remaining 253 articles in accordance with inclusion criteria (see Fig 1). For a full list of these 253 articles, and reasons for exclusion please see Supplementary Material S1 Table. Twenty-three articles met (or were close to meeting) inclusion criteria, and are described in Table 1.

**Table 1:** Characteristics of all studies included in meta-analysis. All data were extracted by CS and FM in July 2024. All studies meet eligibility criteria.

Article	n (total or used to calculate effect size if different)	Population	Location	Age (central tendency)	Percentage of sample that were female	Covariates included in analysis	Joanna Briggs Quality Assessment score (%)	Masculinity measure	Suicidality measure	Pearson’s r
Coleman (2015) (data from 2001) [32]	2431	University students	US	~18.5	65	Sex, ethnicity, LGBTQ, parental violence, childhood sexual abuse, depression, substance misuse	100	Extended Personal Attributes Questionnaire, including scores for “positive masculinity” and “traditional masculinity” (Helmrich et al., 1981) [63]	Suicidal ideation using 2 items (thoughts of suicide and hopelessness)	- Positive masculinity −0.11 - Traditional masculinity 0.17
Coleman et al., 2020 [55] (see also Feigelman et al., 2021) [64])	10210	Cohort followed over 15 years (first 4 waves of National Longitudinal Study of Adolescent Health (Add Health, 2014))	US	~35	0		100	Traditional masculinity (Cleveland’s 16 gender-discriminating items, 2001). Participants scoring above 73% probability of being male on latent gender-discriminating variable treated as high in traditional masculinity [65].	Cause of death (using National Death Index procedures): suicide or other	0.02
									Suicidal ideation in the last year (yes or no, taken from questions included in the ADD Health survey, 2014) [66]	−0.05
									Suicide attempt(s) in the last year (yes or no, taken from questions included in the ADD Health survey, 2014)) [66]	−0.01
Daruwala et al., 2021 [60]	953	Military personnel	US	27.06	17.7	Age, sex, ethnicity, education, marital status, annual household income, deployment status, aggression, sensation seeking,	100	Self reliance sub-scale of the Conformity to Masculine Norms Inventory (Mahalik et al., 2003) [67]	Acquired Capability of Suicide Scale – Fearlessness about Death (Ribeiro et al., 2014) [68]	−0.07
								The Liverpool Stoicism Scale (Wagstaff & Rowledge, 1995) [69]		0.24
Easton et al., 2013 [56]	487	Victims of childhood sexual abuse	US	50.4	0		100	Conformity to masculine norms (Conformity to Masculine Norms Inventory; Mahalik et al., 2003) [67]	Suicidal ideation item from the GMDS (Dennis et al., 2007) [70]	0.04
Fadoir et al., 2020 [71]	79	Patients hospitalised for recent suicidality	US	38.89	100		100	Restricted emotionality subscale of Conformity to Masculine Norms Inventory (Mahalik et al., 2003) [67]	Ideation in last 48 hours (Modified Scale for Suicide Ideation; Miller et al., 1986) [72]	0.24
	106				0					0.08
Genuchi (2019) [73]	94	Homeless people	US	44.92	0		100	Conformity to masculine norms (Conformity to Masculine Norms Inventory; Mahalik et al., 2003) [67]	Ideation (Beck Scale for Suicidal Ideation; Beck & Steer, 1993) [74]	0.2
								Winning subscale		0.03
								Emotional control subscale		0.1
								Risk taking subscale		0.01
								Violence subscale		0.33
								Power over women subscale		0.07
								Playboy subscale		0.4
								Self reliance subscale		0.25
								Primacy of work subscale		−0.02
								Heterosexual presentation		−0.2
Genuchi et al., 2024 [75]	785	Participants who had experienced stressful recent life events	US	37.78	0		100	Male Role Norms Inventory – Short Form (Levant, Hall, & Rankin, 2013), Self-reliance Through Mechanical Skills subscale [76]	Total suicidality from revised SBQ-R (Osman et al., 2001) [77]	0.2
								Male Role Norms Inventory – Short Form (Levant, Hall, & Rankin, 2013), Toughness subscale [76]		0.25
Granato et al., 2015 [78]	545	Undergraduate students	US	20.27	61.1		100	Gender Role Conflict Scale (O’Neill et al., 1986) total [79]	Acquired Capability for Suicide Scale (Van Orden et al., 2008) [80]	0.15
								Success, Power, and Competition subscale		0.15
								Restrictive Emotionality subscale		0.14
								Restrictive Affectionate Behavior Between Men subscale		0.13
								Conflict Between Work and Family Relationships subscale		<0.01
Hobbs & McLaren, 2009 [81]	159	Older adults	Australia	73.09	0		100	Agency (EPAQ. Spence et al., 1974) [63]	Ideation (General Health Questionnaire; Goldberg and Hillier, 1974) [82]	−0.26
					100					−0.35
Houle et al., 2008 [18]	80	Participants with recent stressful life events	Canada	20-59	0		100	Gender Role Conflict Scale (O’Neil et al., 1986) [79]	Suicide attempt (hospital admission records)	0.43
								Success, power and competition subscale		0.31
								Restrictive affectionate behavior between men subscale		0.36
								Work-family conflict subscale		0.12
								Restricted emotionality subscale		0.42
Hunt et al., 2006 (data collected in 1995/6) [21]	676	Cohorts of community members from West of Scotland	UK	23	52.76	Sex	87.5	Gender role orientation using the Short Form of the Bem Sex Role Inventory (Bem, 1974) [63]	Suicidal ideation (single item measure: “Have you ever had serious suicidal thoughts?”	0.01
	754			43	56.04					−0.11
	723			63	55.42					−0.06
Jacobson et al., 2011 (data collected in 2005) [83]	83	High school students	US	14.8	41.6		100	Restrictive Emotionality Subscale of Adolescent Gender Role Conflict Scale (Blazina et al., 2005) [84]	Suicidal ideation in the last month (Suicidal Ideation Questionnaire;Reynolds, 1988) [85]	0.16
	105								Lifetime history of suicide attempt (Diagnostic Interview Schedule for Children, Shaffer et al., 2000) [86]	0.15
King et al., 2020 [57]	829	Australian Longitudinal Study on Male Health (Ten to Men Study)	Australia	~17.5	0		100	Conformity to masculine norms (Conformity to Masculine Norms Inventory; Mahalik et al., 2003) [79]	Ideation in last year (Youth Risk Behavior Survey, 2011) [87]	−0.02
Kopper et al., 2001 [88]	139	Students	US	~19	100		100	Masculinity (Minnesota Multiphasic Personality Inventory-2, Butcher, 2010) [89]	Ideation (Suicide Probability Scale, Cull & Gill, 1982) [90]	0.08
	75				0					0.37
Lee et al., 2020 (data collected 2006–2016) [58]	125	Psychiatric inpatients and outpatients with Borderline Personality Disorder diagnosis	Korea	28.39	56.3		100	Masculinity (Minnesota Multiphasic Personality Inventory-2, Butcher, 2010) [89]	History of suicide attempts (from medical records)	−0.05
O’Beaglaoich et al., 2020 [91]	176	Adolescents	Ireland	16.9	0		100	Gender role conflict (Irish Gender Role Conflict Scale for Adolescents; O’Beaglaoich et al., 2016) [91]	Ideation in past 2 weeks (negative subscale of Suicide Ideation Scale; Osman et al., 1998) [77]	0.43
Perry et al., 2019 [61]	149	Undergraduate students	US	20.14	53		100	Liverpool Stoicism Scale (Wagstaff & Rowledge, 1995) [69]	The Acquired Capability for Suicide Scale (Van Orden et al., 2008) [80]	0.31
Rice et al., 2020 [58]	530	Online survey	Canada	47.91	0	Population was sampled to represent the community on socioeconomic status and age	100	Masculine values (Intensions Masculine Values Scale (Oliffe et al., 2019)) [92]	Lifetime ideation (revised SBQ-R (Osman et al., 2001)) [77]	−0.08
									Ideation in the past year (revised SBQ-R (Osman et al., 2001)) [77]	−0.07
									Previously disclosed ideation (revised SBQ-R (Osman et al., 2001)) [77]	−0.16
									History of suicide attempt (revised SBQ-R (Osman et al., 2001)) [77]	−0.03
Straiton et al., 2012 [33]	309	Online survey	Norway	25.5	100		100	Instrumentality (Australian Sex Role Scale; Antill et al., 1981) [93]	Ideation in last 2 weeks (BDI item 9; Beck & Steer, 1984) [94]	−0.36
								Unmitigated agency (Australian Sex Role Scale; Antill et al., 1981) [93]		−0.12
	172				0			Instrumentality (Australian Sex Role Scale; Antill et al., 1981) [93]	Ideation in last 2 weeks (BDI item 9; Beck & Steer, 1984) [94]	−0.33
								Unmitigated agency (Australian Sex Role Scale; Antill et al., 1981) [93]		−0.12
Waelde et al., 1994 [95]	292	Undergraduate students	US	~19	100	Femininity, achievement events, and interaction terms	100	Personal Attributes Questionnaire (Spence & Helmreich, 1978) [63]	Suicidal ideation (Beck Depression Inventory item 8) [94]	−0.54
	238				0					−0.38
Walther et al., 2023 [54]	490	Online community participants	Germanspeaking European countries	25.7	0		87.5	Male Role Norms Inventory – Short Form (Levant, Hall, & Rankin, 2013) [76]	Self-reported suicide attempts in lifetime	0.02
									Self-reported suicide attempts in last month	0.06
									Suicidal ideation in past 2 weeks	−0.01
Wang et al., 2018 [53]	500	LGBTQ men	Taiwan	22.9	0	Age, education, parents’ education, homosexuality, age at coming out, peer and family support, bullying	87.5	Self-rated masculinity during childhood and adolescence (single item)	Suicidality composite measure including ideation and action (Kiddie-SADS suicide items; Puig-Antich & Chambers, 1978) [96]	−0.07
Witte et al., 2012 [97]	185	Undergraduate students	US	18.7	38		100	Liverpool Stoicism Scale (Wagstaff & Rowledge, 1995) 81)	Acquired Capability for Suicide Scale (Van Orden et al., 2008) fearlessness of death subscale [80]	0.11
	378			19.7	52					0.12

Insufficient statistical information was available to include three further studies which otherwise met inclusion criteria [50–52].

All studies scored 100% on the JBI quality appraisal tool, with the exception of the following. Two studies failed to meet inclusion criteria on the basis of having used single self-report measures of (a) childhood masculinity (i.e.,self-rated level of masculinity during childhood and adolescence from 1 (very low) to 5 (very high) [53] and (b) experience of suicidality (i.e., “have you ever seriously thought about taking an overdose of drugs or injuring yourself deliberately?”), [21], and two results from one further study [54] had also utilised single self-report measures of suicidality for which measures of validity and reliability were not available (i.e., self-reported numbers of suicide attempts in last month and lifetime). To be inclusive, however, we conducted analyses with and without these results included.

A variety of measures of effect size and magnitudes of relationships were reported in the studies included in analyses (Pearson’s r, odds ratios, R^2^, and beta). Since our research questions concerned relationships between masculinity and suicidality, it is perhaps not surprising that the majority of studies identified in our search reported relationships between continuous variables (n = 13). Where outcomes were dichotomous, the variable was presence or absence of suicidal ideation or action [18,21,33,53–59]. Where continuous data (e.g., on suicidal ideation) had been collected but not reported, we contacted authors to request access to this, or for access to Pearson’s r based on the continuous data. These were provided in four cases [21,53,56,57]. Rather than excluding studies for which the outcome variable was dichotomous, and risk incorporating a source of bias into our analyses, we converted all other measures of effect size to Pearson’s r. We treated beta as equivalent to r [32,60,61]. While the importance of justification for converting effect sizes has been emphasised, conversions maintain fidelity to the original effect size [62].

### Grouping of variables

Table 1 shows characteristics and effect sizes for individual studies.

#### Location.

Geographically, the greatest proportion of samples were from the USA (n = 13 studies or 57%). Two were from Canada and two from Australia, and one each were from the UK, Ireland, Norway, Korea, German-speaking European countries, and Taiwan. Given the small number of studies from countries other than the US, we grouped this measure as North America (USA and Canada, n = 15), Europe (UK, Ireland, Norway, and German-speaking European n = 4), Asia (Korea and Taiwan, n = 2) and Australia (n = 2), and also as North America (n = 15) and other (n = 8).

#### Demographic characteristics.

Sample age ranged from young to older adults. Age was treated as a continuous variable (see Methods and Table 1).

In all studies participants were described as either ‘male’ or ‘female’ without specifying whether this reflected sex designated at birth, gender identification, or other. Therefore, while there may be exceptions, we have made the assumption that participants across studies have been assigned a label based on their self-reported sex, or information included in their medical or death records, and that this is likely to reflect sex designated at birth in the majority of cases. We acknowledge the significant limitations of this categorisation in the context of the complexity and nuance of gender identification, and explore this further in the Discussion.

Ten studies included only male participants (44%), and the rest included both men and women. Percentage of the sample that was female was treated as a continuous variable (see Methods).

Eleven studies reported ethnicity of participants, and in all cases this was expressed as the percentage of the sample which was white. Mean percentage of sample which was white for these studies was 76%.

#### Measure of masculinity.

Across the 23 studies, 11 different masculinity assessment tools were implemented, introducing considerable methodological heterogeneity.

The measures that were most commonly used were the Conformity to Masculine Norms Inventory (CMNI [67]: n = 5 studies, 24%) and the Gender Role Conflict Scale (GRCS [79]: n = 4 studies, 19%). The CMNI assesses the extent to which individuals conform to the actions, thoughts, and feelings consistent with masculine norms in dominant culture in the US at the time of development [98]. It comprises eleven distinct factors: Winning, Emotional Control, Risk-Taking, Violence, Dominance, Playboy, Self- Reliance, Primacy of Work, Power Over Women, Heterosexual Presentation, and Pursuit of Status. Validity and internal consistency of the scale and its eleven subscales have been shown across a number of samples of largely white, heterosexual, undergraduate student men, and it has demonstrated good differential validity in comparing women and men, as well as men on health related variables [67,99]. The GRCS was developed in the 1980s on two undergraduate male samples, and assesses adherence to “success, power and competition”, “restrictive emotionality”, “restrictive affectionate behavior between men”, and “conflict between work and family”. A validation was conducted in 2018 [92] in which calculation of overall gender role conflict using all 16 items was contraindicated.

One study utilized a measure of masculinity explicitly conceptualized as having positive impacts on health. The Masculine Values Scale (MVS [100]) was developed using samples of Canadian men and assesses strength-based masculinity along two domains: open and selfless, and healthy and autonomous. Scores on this scale have been found not to correlate with measures of traditional masculinity [100]. Therefore, while we included this study in meta-analysis, we also conducted analyses without this included since it may not fit with the ‘traditional masculinity’ framework.

Smaller numbers of studies utilized further measures of masculinity. The Liverpool Stoicism Scale [69] was used in three studies, and assesses stoicism through lack of emotional involvement, dislike for openly expressing emotion, and the ability to withstand emotion. We considered this to be consistent with Houle et al.’s, (2008) [18] and Jansz’s (2000) [19] definition of traditional masculinity (see Introduction) so included these studies in our analyses. The EPAQ was also used in three studies, and this measures adherence to psychological traits judged to be more typical of males than females along ‘positive’ (e.g., self confidence, decisiveness) and ‘negative’ (e.g., dominant, aggressive) dimensions [63]. Again, we considered this measure to be consistent with traditional masculinity and included these studies in analyses. Single studies used different measures of masculinity (see Table 1), all of which were considered to sit within a broad ‘traditional masculinity’ construct.

As there were small numbers of studies which used each measure of masculinity, we grouped studies into the following categories for meta-regression: those using the CMNI (n = 5); those using the GRCS (n = 4); those using the Liverpool Stoicism Scale (n = 3); those using the EPAQ (n = 3); those using other measures of masculinity (i.e. Cleveland’s 16 gender-discriminating items, 2001 [65]; the Bem Sex Role Inventory (Bem, 1974) [101]; Minnesota Multiphasic Personality Inventory-2 (1989) [89]; Australian Sex Role Scale (Antill et al., 1981) [93]; self-rated childhood masculinity; The Male Role Norm Inventory (Levant et al., 2013) (n = 14) [76]; and that using the MVS (n = 1) [100].

#### Measure of suicidality.

Suicidality was similarly assessed in a variety of ways. As many studies included more than one measure of suicidality, the following reports numbers of results (rather than numbers of studies) which utilised each measure. Suicidal ideation (i.e., thoughts of suicide) over various timeframes from the last 48 hours to lifetime was the most commonly used measure (in 39, or 65% of, results). A history of suicide attempts was utilised in eleven (18%) of results. One (2%) result was based on cause of death. Ten (16% of results) assessed acquired capability for suicide, which includes factors theorised to allow individuals to develop the ability to engage in a suicidal act based on Joiner’s (2005) Interpersonal Theory of Suicide [102,80]. These include heightened fearlessness and reduced pain sensitivity [102,80]. While acquired capability is not necessarily an indication of suicidal intent, it is a risk factor for suicide, and we chose to include it here in order to explore its relation to masculinity. We grouped studies into the following categories: suicidal ideation, suicidal action including a history of suicide attempts and deaths by suicide, and acquired capability for suicide. Since acquired capability is conceptually distinct from our other measures of suicidality, where we grouped measures of suicidality together we have reported results with and without acquired capability included.

### Meta-analysis and meta-regressions

#### RQ1: Are psychometric measures of traditional masculinity correlated with suicidality?.

**Fig 2:**
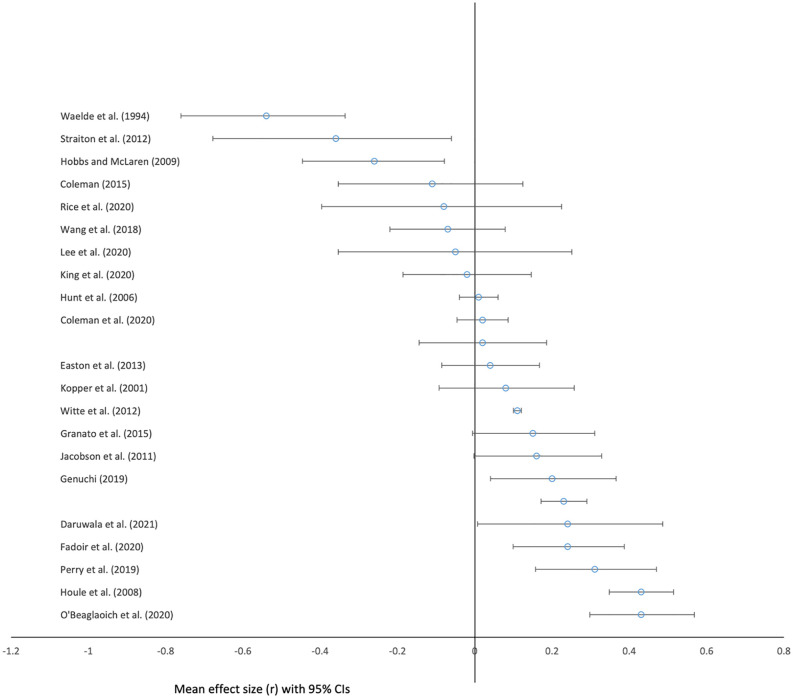
Forest plot showing mean effect sizes (r) and 95% confidence intervals for all 23 studies testing relationships between masculinity and suicidality.

**Fig 3:**
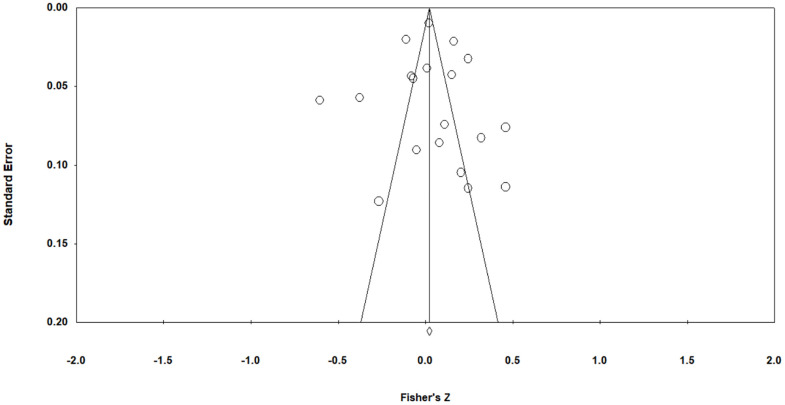
Funnel plot testing for publication bias.

The mean pooled effect size across all 23 samples was small, positive, and non-significant (r = 0·03 [95% CI: −0·01, 0·1], z = 1.75, p = 0.341). See Fig 2. There was significant heterogeneity (I^2^ = 92.2, Q [23] = 294.69, p < 0·001). Duval and Tweedie’s Trim and Fill method estimated 3 studies to be missing to the left of the mean. With this imputed, the effect size was 0.01 (95% CI: −0·001, 0·03). For funnel plot see Fig 3.

With one study removed at random, the result was largely the same (r = 0·04 [95% CI: −0·01, 0·08], z = 1.51, p = 0.13).

With Rice et al. (2020), Wang et al. (2018), Walther et al. (2023), and Hunt et al. (2006) excluded, the relationship remained small and positive, and reached statistical significance (r = 0·06 [95% CI: 0·02, 0·1], z = 2.71, p = 0.007).

With studies using measures of acquired capability for suicide excluded, the relationship remained small and positive (r = 0·04 [95% CI: −0·01, 0·07], z = 2.12, p = 0.14).

Joanna Briggs quality scores were high for all studies (100% in all but 3 cases, for which the score was 87.5%; Hunt et al. (2006), Wang et al. (2019), and Walther et al. (2023)). See Table 2. With these three studies excluded, the relationship remained small, positive and non-significant (r = 0·05 [95% CI: −0·004, 0·1], z = 1.81, p = 0.07).

**Table 2:** Item scores for Joanna Briggs Critical Appraisal Tool for Cross-Sectional Studies.

Study	JB1 Were the criteria for inclusion in the sample clearly defined?	JB2 Were the study subjects and the setting described in detail?	JBI3 Was the exposure measure in a valid and reliable way	JBI4 Were objective, standard criteria used for measurement of the condition?	JBI5 Were confounding factors identified?	JBI6 Were strategies to deal with confounding factors stated?	JBI7 Were the outcomes measured in a valid and reliable way?	JBI8 Was appropriate statistical analysis used?	Total JBI
Coleman (2015)	1	1	1	1	1	1	1	1	8
Coleman et al. (2020)	1	1	1	1	1	1	1	1	8
Daruwala et al. (2021)	1	1	1	1	1	1	1	1	8
Easton et al. (2013)	1	1	1	1	1	1	1	1	8
Fadoir et al. (2020)	1	1	1	1	1	1	1	1	8
Genuchi (2019)	1	1	1	1	1	1	1	1	8
Genuchi et al. (2024)	1	1	1	1	1	1	1	1	8
Granato et al. (2015)	1	1	1	1	1	1	1	1	8
Hobbs & McLaren (2009)	1	1	1	1	1	1	1	1	8
Houle et al. (2008)	1	1	1	1	1	1	1	1	8
Hunt et al. (2006)	1	1	0	1	1	1	1	1	7
Jacobson et al. (2011)	1	1	1	1	1	1	1	1	8
King et al. (2020)	1	1	1	1	1	1	1	1	8
Kopper et al. (2001)	1	1	1	1	1	1	1	1	8
Lee et al. (2020)	1	1	1	1	1	1	1	1	8
O’Beaglaoich et al. (2020)	1	1	1	1	1	1	1	1	8
Perry et al. (2019)	1	1	1	1	1	1	1	1	8
Rice et al, (2020)	1	1	1	1	1	1	1	1	8
Straiton et al. (2012)	1	1	1	1	1	1	1	1	8
Waelde et al. (1994)	1	1	1	1	1	1	1	1	8
Walther et al. (2023)	1	1	1	1	1	1	0	1	7
Wang et al., 2018 [53]	1	1	0	1	1	1	1	1	7
Witte et al. (2012)	1	1	1	1	1	1	1	1	8

#### RQ2: Do different operationalisations of traditional masculinity (or components of traditional masculinity) show different relationships (in terms of direction and magnitude) with suicidality?.

Across all 61 results from 23 studies, the relationship was small, positive, and non-significant (r = 0·05 [95% CI: −0·01, 0·1], z = 3.07, p = 0.083). Measure of masculinity significantly moderated the relationship (Q(4,55) = 635.89, p < 0.001).

**Fig 4:**
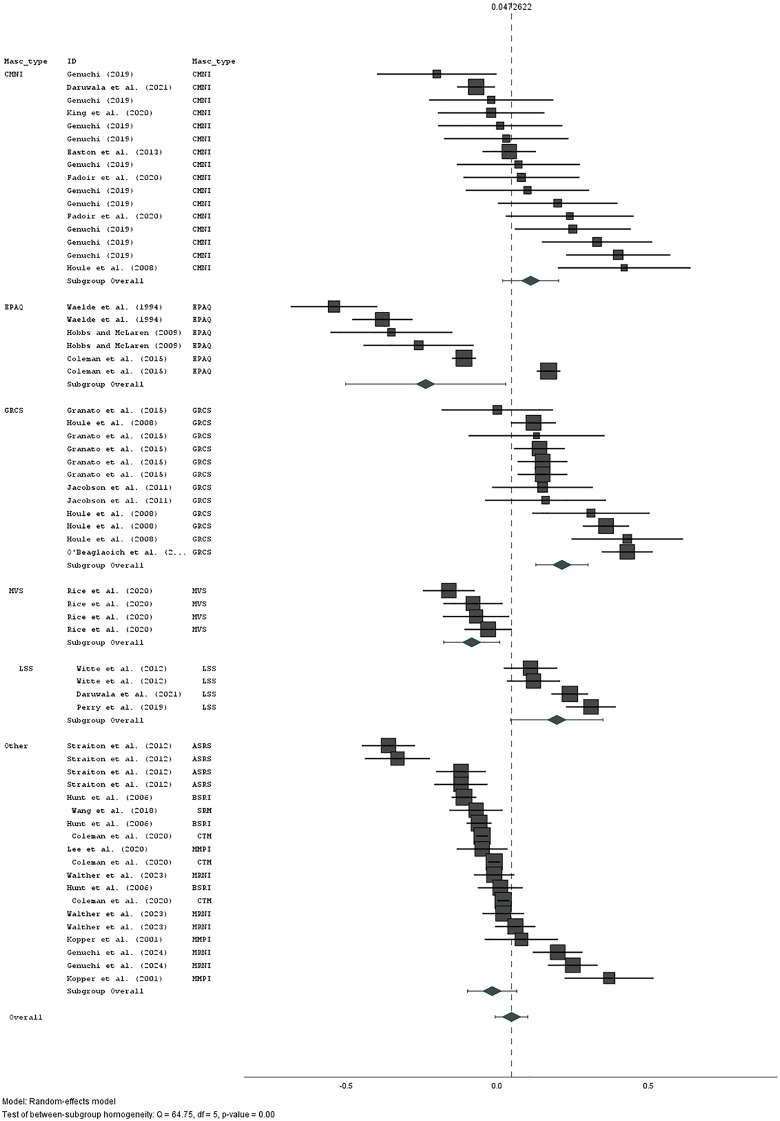
Significant moderation of relationship between measures of masculinity and suicidality by measure of masculinity. CMNI: Conformity to Masculine Norms Inventory [67]; EPAQ: Extended Personality Questionnaire [63]; GRCS: Gender Role Conflict Scale [79]; MVS: Masculine Values Scale [92]; LSS: Liverpool Stoicism Scale [69]; Other: all other measures of traditional masculinity including CTM [65], BSRI [101], MMPI [89] ASR [93], SRM (self-rated childhood masculinity; 53), MRNI [76].

There were small positive significant relationships between suicidality and CMNI (r = 0·11 [95% CI: 0·03, 0·18], z = 2.65, p = 0.008), GRCS (r = 0·18 [95% CI: 0·13, 0·23], z = 6.7, p < 0.001), and stoicism (r = 0.2 [95% CI: 0.11, 0.28], z = 4.36, p < 0.001). There were small, negative, significant relationships between suicidality and EPAQ (r = −025 [95% CI: −0.45, −0.03], z = −2.22, p = 0.026), and MVS (r = −0.09, [95% CI: −0.14, −0.03], p = 0.002). The relationship between suicidality and other psychometric measures of masculinity was small, negative, and non-significant (r = −0.03 [95% CI: −0.08, −0.03], p = 0.334). See Fig 4.

#### RQ3: Does masculinity correlate differently (in terms of direction and magnitude) to suicidal ideation, suicide attempts, and deaths by suicide?.

**Fig 5:**
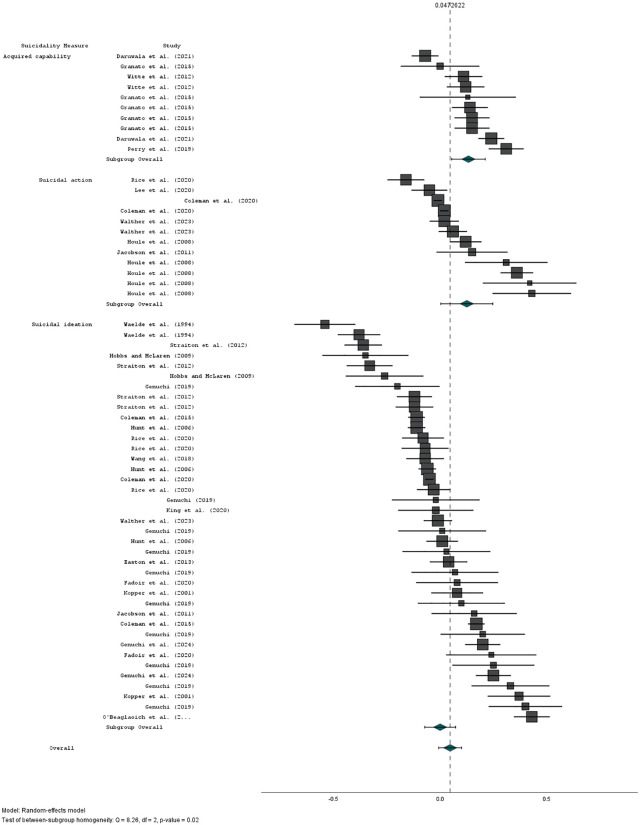
Significant moderation of the relationship between suicidality and measures of masculinity by measure of suicidality.

Across all 61 results from 23 studies, measure of suicidality significantly moderated the relationship between masculinity and suicidality (F(2,60) = 3.33, p = 0.0443, R^2^ analog = 0.1). There were small, positive, significant relationships between psychometric measures of masculinity and acquired capability (r = 0.13 [95%CI: 0.05, 0.21], z = 3.82, p = 0.004), and suicidal action (r = 0.13 [95%CI: 0.01, 0.25], z = 2.26, p = 0.045). The relationship between psychometric measures of masculinity and suicidal ideation was non-significant (p = 0.993). See Fig 5.

#### RQ4: Are relationships between measures of traditional masculinity and suicidality moderated by personal characteristics (age, sex, or ethnicity), or local context (country)?.

Across all 61 results from 23 studies, percentage of the sample that was female, age, location, and percentage of the sample that was white did not significantly moderate the relationship between psychometric measures of masculinity and suicidality (all p > 0.1).

## Discussion

Here we have reported results of the first meta-analysis testing the relationship between measures of masculinity and suicidality. Across 23 studies we found no evidence of a consistent significant relationship. This is likely due, at least in part, to significant heterogeneity in measures of both masculinity and suicidality, but may also reflect greater complexity and nuance than can be captured in a straightforward linear relationship between two broad constructs. We argue, then, that our analysis of the relationship between masculinity and suicidality overall should not be interpreted as a meaningful or reliable indication of any link between components of the two. Meta-regression results supported this by demonstrating significant moderation of the relationship by measure of masculinity and by measure of suicidality. In other words, the relationship between measures of masculinity and suicidality depends upon both the specific measure of masculinity as well as the specific measure of suicidality, emphasising the importance of treating ‘masculinity’ as an overarching umbrella term to describe a wide range of constructs. The results of our meta-regression analyses should be treated as the primary outcome of our study, then, as they demonstrate the diversity of conceptualisations and components of masculinity and the variety of ways in which they interact with suicidality.

The relationship between suicidality and measure of masculinity was significantly moderated by measure of masculinity. There were small, positive, significant relationships between suicidality and three measures of masculinity (CMNI, GCRS, and stoicism). There were small, negative, significant relationships between suicidality with the EPAQ and the MVS. The relationship between the grouping of other measures of traditional masculinity with suicidality was non-significant. While being cautious not to state firm conclusions based on these results, given the relatively small numbers of studies which utilised each measure, it is possible that looking more closely at the scales which do and do not correlate with suicidality can tell us something about the nuance. The CMNI [67] specifically assesses conformity to masculine norms in the US. Its domains (Winning, Emotional Control, Risk-Taking, Violence, Dominance, Playboy, Self-Reliance, Primacy of Work, Power Over Women, Disdain for Homosexuals, and Pursuit of Status) can be broadly linked to pursuit of status and emotional control. Therefore, it is likely to be linked conceptually and empirically to stoicism. Stoicism was measured in the studies included here using the Liverpool Stoicism Scale [69] which assesses lack of emotional involvement, dislike for openly expressing emotion, and the ability to withstand emotion. The GRCS [79] was designed specifically to assess the negative consequences of gender role conformity. Its measures are similar to those of the CMNI in relation to pursuit of status (Success, power, competition) and to both the CMNI and stoicism in terms of restrictive emotionality, as well as extending these to conflicts between work and family relations, homophobia, and public embarrassment from gender-role deviance. Perhaps, then, the form of traditional masculinity associated with increased suicidality is that which is concerned with pursuit of status and restricted emotionality.

Those measures of masculinity for which the converse relationship with suicidality was found (i.e., higher masculinity was associated with reduced suicidality) were the MVS and the EPAQ. The MVS [92] stands out in our sample as (a) being significantly more modern than most others, and (b) as being designed specifically to assess strength-based masculinity. As it has been found not to correlate with traditional masculinity [100], we reasoned that this may not assess traditional masculinity and conducted analyses with and without this measure included (its removal had little impact on the magnitude of the relationship). This was supported by the negative relationship with suicidality, suggesting these strength-based traits to be protective against suicidality. The negative relationship between the EPAQ [63] and suicidality may stem from its measurement of traits which are considered to be desirable and undesirable masculine characteristics (e.g., self-confidence, assertiveness). It is possible, then, that there are traditional masculine traits which are protective against suicide, as well as those which increase risk.

That there was no relationship between our grouping of ‘other’ psychometric measures of masculinity with suicidality may be due to the diversity of measures included, each of which was associated with a single or very small number of studies, but also to a tendency to measure both desirable and undesirable, or positive and negative, masculine traits. The Australian Sex Role Scale [93], for example, is comprised of items that are socially desirable and undesirable for men and women. The study which used this measure [33], used one desirable (instrumentality; i.e., having agency in accomplishing tasks) and one non-desirable trait (unmitigated agency; i.e., a significant focus on the self and one’s own achievements and a negative view of others). The Bem Sex Role Inventory [101] similarly measures psychological traits considered socially desirable and undesirable for men and women. Some of these are consistent with those included in the measures of traditional masculinity described above (e.g., dominant, aggressive, competitive) whereas others are perhaps more closely linked to positive health outcomes (e.g., being analytical, assertive, and making decisions easily). The MMPI masculinity-femininity scale [89] reflects gender-related psychopathology, so may not tap into the same constructs of underlying masculinity as other scales. Finally, Cleveland’s (2001) gender-discriminating attitudes and behaviours are those used to distinguish male- from female-identifying individuals [65]. Again, these items may include those that are both detrimental to (e.g., not emotional), and protective of (e.g., liking oneself), health. Therefore, it is possible that the combination of results using these measures did not converge on specific elements of traditional masculinity which can lead to development of suicidality. These findings emphasise the nuance in the relationship between masculinity and suicidality, and suggest that some traits (e.g., emotional control and pursuit of status) are associated with risk of suicidality, whereas others (e.g., ‘positive’ components of traditional masculinity, and contemporary strength-based masculinity) may be protective against suicidality.

Measure of suicidality significantly moderated the relationship between measures of masculinity and suicidality, such that there were small positive relationships between psychometric measures of masculinity and suicidal action and acquired capability for suicide. The relationship with suicidal ideation was non-significant.

Acquired capability is the capability to engage in a suicidal act [102,80] which is developed over time and includes fearlessness of death and pain insensitivity via painful and aversive life experiences. Therefore, this result might tell us that those who score more highly on psychometric measures of traditional masculinity are more frequently exposed to such experiences and/or have used such experiences in a way which increases capability for suicide differently to those who score lower on such measures of masculinity.

The studies included in the meta-analysis which we grouped as ‘suicidal action’ included a history of suicide attempts and deaths by suicide. There were too few studies which utilised deaths by suicide for us to include this as a separate grouping, meaning it was not possible to determine whether psychometric measures of masculinity were differentially associated with suicide attempts versus deaths by suicide, thus limiting our ability to conclude that our results inform our understanding of the gender paradox of suicide. However, they do suggest a stronger relationship with suicidal action than ideation, which is in support of a role of traditional masculinity in explaining the paradox.

It is important to take into consideration, however, the possibility that there was systematic variation in the validity of measures of suicidal ideation versus action. Measures of action, for example, included more objective items (e.g., cause of death, hospital admission records) whereas all measures of ideation were self-reported. It is possible that the variation inherent in self report data (e.g., data collected at a time point distal to that of the experience of suicidal ideation, or difficulties in remembering distressing emotions) led to smaller effect sizes. Furthermore, there is the potential for a desirability bias effect such that men may be less likely than women (or, more masculine individuals more likely than less masculine individuals) to report suicidal ideation. For example, in high income countries such as those for which the gender paradox has been reported, suicide attempts and ideation, and deaths by suicide, are considered to be gendered behaviours. Death by suicide is considered masculine and suicide attempts and ideation considered feminine [16]. Given the potential for societal punishment of individuals who diverge from gendered social norms (e.g., [103–105]), the fear men have of being judged by their peers and receiving this kind of criticism may alone be substantial enough to pressure them into conforming [106]. Therefore, it is possible that data on suicidal ideation in more masculine individuals represent an underestimation.

Here we did not find relationships between measures of masculinities and suicidality to be moderated by context (i.e., region) or individual characteristics (i.e., age, ethnicity or gender). We acknowledge, however, the limited number of studies in areas outwith North America, the small number of studies which reported ethnicity, and the relatively small sample size overall, which may have obscured any intersection with these variables. There is good evidence from the wider literature that traditional masculine characteristics, suicidality, and the relationship between the two, develop and are enacted differently across contexts such as age (e.g., [34]) and rurality [40,107,108]. Therefore, while we failed to find specific differences across contexts, it may be that the measures employed in the studies included were too blunt, and that our questions did not support detailed analysis of masculinity and suicidality in their full complexity.

Heterogeneity was introduced into our analyses via a number of sources. These included those described above in terms of diverse measures of suicidality and masculinities, but also included varied clinical and demographic sample characteristics, including non-clinical (e.g., undergraduate students, and participants in online surveys or cohort studies) and clinical (e.g., those hospitalised for suicide attempts, and psychiatric patients) as well as specific populations including military, those experiencing homelessness, adult survivors of childhood sexual abuse, LGBTQ men, and those experiencing recent life stress. At the same time, however, our sample was heavily biased towards high income westernised societies. As argued above, further work which seeks to explore masculinity and its impact upon suicidality in local contexts would be advantageous, as would work testing alternative constructions of masculinities and their link to suicidality.

A potential statistical limitation of our method of meta-regression was inclusion of multiple effect sizes within a single study, violating assumptions of independence. Although modern methods are robust to this if corrected for repeated sampling using the Hartung-Knapp correction [49], as we ensured here, it is prudent to mention this potential statistical issue here.

There were significant time lapses between the development of many of the masculinity measures (e.g., [67,69,79]) and assessment of suicidality in a number [79] of the studies included in our analyses. It is likely that our cultural understanding of masculinity has evolved in the decades since [69] the development of such measures. Contemporary masculinities are currently poorly understood, and likely to be rapidly evolving (e.g., [109]) and the development of measures would allow systematic evaluation of its components and impacts on health. Relatedly, gender identification is a rapidly developing field in high income westernised societies. Here we assumed ‘sex’ of participants of the studies we included to be sex designated at birth. We acknowledge the significant limitations of this categorisation in the context of the complexity and nuance of gender identification and hope that future work will be able to link the growing diversity of gender identifications and disentanglement from designated sex at birth, on suicidality.

Finally, a focus on masculinity in relation to larger rates of deaths by suicide amongst men may neglect to identify protective effects of femininity. For example, elements of the traditional female role (such as concern for others who are left behind, or use of less lethal methods to avoid disfigurement or distressing experiences for those who find the body) prevent deaths by suicide.

## Conclusion

Here we report no clear relationship between diverse measures of masculinity overall and suicidality. We argue that it is likely to be meaningless to interpret this finding given the significant heterogeneity in operationalisations of masculinity and suicidality in the studies included in the analysis. However, we found nuanced effects which suggest that it is perhaps specifically the pursuit of status and emotional restriction that increase the risk of suicidality. Others, including positive traits of traditional masculinity and strength-based masculinity may be protective against suicidality. Furthermore, masculinity may have a stronger impact on suicidal action than ideation, although this result may stem from under-reporting of suicidal ideation by those who score more highly on measures of traditional masculinity. We suggest that the more complex and nuanced interactions of different components and conceptualisation of masculinity with suicidality should be treated as the core outcome of our study. We argue for the importance of exploring potential protective functions of femininity as well as broader conceptions of gender roles.

## Supporting information

S1 Table A list of the articles where abstracts were screened in accordance with inclusion criteria, and reasons for exclusion. (XLSX)

S1 Appendix Prisma Checklist. (DOCX)

S2 Appendix Literature searches. (DOCX)
