# Supplementary material for: Masculinities and suicide: A systematic review and meta-analysis
Source: PLoS One. 2026 Feb 25;21(2):e0342172. doi: 10.1371/journal.pone.0342172 (PMC12935202; doi:10.1371/journal.pone.0342172)
Supplement: S2 Appendix — (DOCX) [file pone.0342172.s003.docx]

**Appendix B. Literature search methods**

1. We searched the online literature search engine Google Scholar using the following search terms:
2. Allintitle: “gender role”, suicide
3. Allintitle: “gender role”, suicidal
4. Allintitle: “gender role”, suicidality
5. Allintitle: “gender-role”, suicide
6. Allintitle: “gender-role”, suicidal
7. Allintitle: “gender-role”, suicidality
8. Allintitle: “sex role”, suicide
9. Allintitle: “sex role”, suicidal
10. Allintitle: “sex role”, suicidality
11. Allintitle: “sex-role”, suicide
12. Allintitle: “sex-role”, suicidal
13. Allintitle: “sex-role”, suicidality
14. Allintitle: masculine, suicide
15. Allintitle: masculine, suicidal
16. Allintitle: masculine, suicidality
17. Allintitle: masculinity, suicide
18. Allintitle: masculinity, suicidal
19. Allintitle: masculinity, suicidality
20. We searched the online database Web of Science using the following search terms:

(TI = (“gender role*” OR “gender-role*” OR “sex role*” OR “sex-role” OR masculine*) AND (“suicid*” (TOPIC)))

LANGUAGE:(English)
Databases searched: Databases searched: *Arts & Humanities Citation Index, Book Citation Index - Science, Book Citation Index - Social Sciences & Humanities, Conference Proceedings Citation Index, Conference Proceedings Citation Index - Social Science & Humanities, Current Chemical Reactions, Emerging Sources Citation Index, Index Chemicus (IC) - 1993-present, Science Citation Index Expanded, Social Sciences Citation Index, BIOSIS Citation Index, BIOSIS Previews, CABI: CAB Abstracts, Current Contents Connect, Data Citation Index, Derwent Innovations Index, KCI - Korean Journal Database, MEDLINE (1950– present), Russian Science Citation Index, SciELO Citation Index, Zoological Record*.

1. We searched the online database PubMed using the following search terms:

(((“gender role*”[Title/Abstract] OR “gender-role*”[Title/Abstract] OR “sex role*”[Title/Abstract] OR “sex-role*”[Title/Abstract] OR masculine*”[Title/Abstract])) AND (“suicid*”[Title/Abstract]))

1. We searched the online database PsychINFO using the following search terms:
2. KW (“gender role*” OR “gender-role*” OR “sex role*” OR “sex-role” OR masculin*) AND (suicid*)
3. AB (“gender role*” OR “gender-role*” OR “sex role*” OR “sex-role” OR masculin*) AND (suicid*)
4. We conducted forward and backward searches of all articles identified in the first round of screening.
5. We searched journals likely to publish articles on masculinity and suicide (Archives of Suicide Research, International Journal of Men’s Health, Psychology of Men and Masculinity, American Journal of Men’s Health, Journal of Men’s Health, Sex Roles, and Suicidological Research Online).
